# Epidemiological characteristics of COVID-19: a systematic review and meta-analysis

**DOI:** 10.1017/S0950268820001430

**Published:** 2020-06-29

**Authors:** Malahat Khalili, Mohammad Karamouzian, Naser Nasiri, Sara Javadi, Ali Mirzazadeh, Hamid Sharifi

**Affiliations:** 1 HIV/STI Surveillance Research Center, and WHO Collaborating Center for HIV Surveillance, Institute for Futures Studies in Health, Kerman University of Medical Sciences, Kerman, Iran; 2 Department of Biostatistics and Epidemiology, School of Public Health, Kerman University of Medical Sciences, Kerman, Iran; 3 School of Population and Public Health, Faculty of Medicine, University of British Columbia, Vancouver, BC, Canada; 4 Modeling in Health Research Center, Institute for Futures Studies in Health, Kerman University of Medical Sciences, Kerman, Iran; 5 Department of Epidemiology and Biostatistics, Institute for Global Health Sciences, University of California San Francisco, San Francisco, CA, USA

**Keywords:** Coronavirus, COVID-19, epidemiology, pandemic

## Abstract

Our understanding of the Coronavirus disease 2019 (COVID-19) continues to evolve and there are many unknowns about its epidemiology. This study aims to synthesise case fatality rate (CFR) among confirmed COVID-19 patients, incubation period and time from onset of COVID-19 symptoms to first medical visit, intensive care unit (ICU) admission, recovery, and death. We searched MEDLINE, Embase, Google Scholar, and bibliographies of relevant articles from 01 December 2019 to 11 March 2020 without any language restrictions. Quantitative studies that recruited people with confirmed COVID-19 diagnosis were included. Two independent reviewers extracted the data. Out of 1675 non-duplicate studies, 43 were included in the meta-analysis. The pooled mean incubation period was 5.68 (99% confidence interval [CI]: 4.78, 6.59) days. The pooled mean number of days from the onset of COVID-19 symptoms to first clinical visit was 4.92 (95% CI: 3.95, 5.90), ICU admission was 9.84 (95% CI: 8.78, 10.90), recovery was 18.55 (95% CI: 13.69, 23.41), and death was 15.93 (95% CI: 13.07, 18.79). Pooled CFR among confirmed COVID-19 patients was 0.02 (95% CI: 0.02, 0.03). We found that the incubation period and lag between the onset of symptoms and first clinical visit for COVID-19 are longer than other respiratory viral infections including Middle East respiratory syndrome and severe acute respiratory syndrome; however, the current policy of 14 days of mandatory quarantine for everyone potentially exposed to severe acute respiratory syndrome coronavirus 2 (SARS-CoV-2) might be too conservative. Longer quarantine periods might be more justified for extreme cases.

## Introduction

The severe acute respiratory syndrome coronavirus 2 (SARS-CoV-2) was first identified in a few unusual pneumonia patients linked to the Wuhan seafood wholesale market in China in December 2019 [1]. However, it soon grew out of China and the Coronavirus disease 2019 (COVID-19) was declared a pandemic on 11 March 2020, and has been reported in 216 countries, areas, or territories [2]. While the epidemic has slowed down in China due to the strict quarantine and preventive regulations, the numbers of COVID-19 patients (i.e. 10021401 as of 28 June, 2020) and confirmed deaths (i.e. 499913 as of 28 June, 2020) are rapidly increasing [2] and have surpassed that of other viruses in the coronavirus family with similar genomes to SARS-CoV-2. For example, SARS which emerged in 2003, infected 8098 patients and caused 774 deaths across 29 countries. The Middle East respiratory syndrome (MERS) which appeared in 2012, led to 2494 patients and 858 deaths across 27 countries [3–6]. The healthcare systems in many countries such as the USA, Spain, Italy, France, UK, Turkey and Iran have been overwhelmed and struggling with the soaring number of patients [7].

Although our understanding of COVID-19's epidemiology is evolving, it is assumed that SARS-CoV-2 is mainly transmitted via droplets and close contacts with people carrying the virus [2]. However, recent reports have also proposed the possibility of the virus being contracted via various surfaces, gastrointestinal transmission [8] and potentially airborne exposures [2, 9]. Based on the existing evidence, elderly population, those with suppressed immune systems and underlying metabolic, cardiovascular or respiratory diseases are at an increased risk for adverse outcomes; however, recent reports from outside China, point to a considerable risk of severe outcomes among the general adult population (i.e. <65 years old) [10, 11].

As we continue to learn more about COVID-19 and its characteristics, there are many unknowns about its epidemiology such as hospitalisation and recovery-related outcomes that are critical for healthcare system preparedness [12, 13]. For example, the mean number of incubation days for COVID-19 varies greatly across the existing literature ranging from 2.5 [14] to >20 days [15, 16]. Our understanding of time from contracting the disease to recovery or death is even more limited. In this systematic review and meta-analysis, we tried to identify the studies that recruited patients diagnosed with COVID-19 and calculate pooled estimates for several epidemiological and clinical outcomes to help provide an overall picture of the epidemiological characteristics of COVID-19. Findings of this study could help inform the ongoing public health and public policy practices across the world.

## Methods

The details of inclusion criteria and our analytical approach were designed a priori and are documented in Open Science Framework (https://osf.io/a3k94/).

### Literature search

Following the Systematic Reviews and Meta-Analyses (PRISMA) checklist (see Supplementary Table S1) and the Peer Review of Electronic Search Strategies (PRESS) guideline [17, 18], we searched PubMed, Embase and Google Scholar from 1 December 2019 to 11 March 2020 for studies that measured and reported several characteristics of COVID-19 (e.g. incubation period, hospitalisation, death). Search terms were combined using appropriate Boolean operators and included subject heading terms/keywords relevant to COVID-19 (e.g. novel coronavirus, sars-cov2, coronavirus disease). Please see Supplementary Table S2 for our sample search strategy.

### Inclusion criteria

Quantitative studies were included in the review if they reported incubation period of of SARS-CoV-2 as well as time from onset of the symptoms to first medical visit, intensive care unit (ICU) admission, recovery (as defined by studies' authors) or death. Studies were also included if they reported the number of deaths among patients with a confirmed COVID-19 diagnosis. Studies were included in the meta-analysis if they provided data on the above-mentioned outcomes along with their standard error and sample size. Case-reports with a sample size of one were removed from the meta-analysis as they did not provide any dispersion estimate. Studies were not excluded based on language, location or measurement method. Given that this study used secondary data and involved no interaction with humans, no ethics approval was required.

### Study selection

Two authors (SJ and NN) completed the abstract and full-text screening, independently. The full-texts of citations that met our inclusion criteria or were unclear were screened by two independent reviewers (SJ and NN). Disagreements over the inclusion of studies were resolved through discussion or by arbitration with the senior author (HS). Duplicate records were excluded.

### Data extraction

Data were extracted independently by the two authors (SJ and NN) and discrepancies were resolved through discussion or by arbitration with the senior author (HS). Data were extracted on publication date, study type (e.g. cross-sectional, case-series, cohort), location, sample size, as well as patients' age and sex. We also extracted data on exposure history, X-ray and computed tomography scan findings, symptoms and underlying conditions in addition to the main outcomes of interests including the number of deaths among confirmed COVID-19 patients (i.e. case fatality rate [CFR]), incubation period and time from onset of COVID-19 symptoms to first medical visit, ICU admission, recovery, and death.

### Statistical analysis

Meta-analysis was performed using STATA's (V.15.1) metan (for numerical variables) and metaprop (for binary variables) commands. The 95% confidence intervals (CI) for binary variables were computed using the exact binomial method. Heterogeneity between the studies was assessed using both the I^2^ statistic with a cut-off of 50% and the *χ*^2^ test with *P*-value <0.10 [19]. As all results turned out to be significantly heterogeneous, we used random-effects models to calculate the pooled point estimate and 95% CI for the CFR, mean time from onset of COVID-19 symptoms to first medical visit, ICU admission, recovery, and death. For the mean incubation period, we estimated 99% CI. We also conducted a random-effects meta-regression using STATA's metareg command to identify the sources of heterogeneity and explored the effect of study-level covariates where data were available (Supplementary Table S3). Meta-regression was considered when there were at least 10 studies included in the meta-analysis [20]. A two-sided *P*-value <0.05 was considered as statistically significant.

## Results

### Participants and study characteristics

**Fig. 1.**
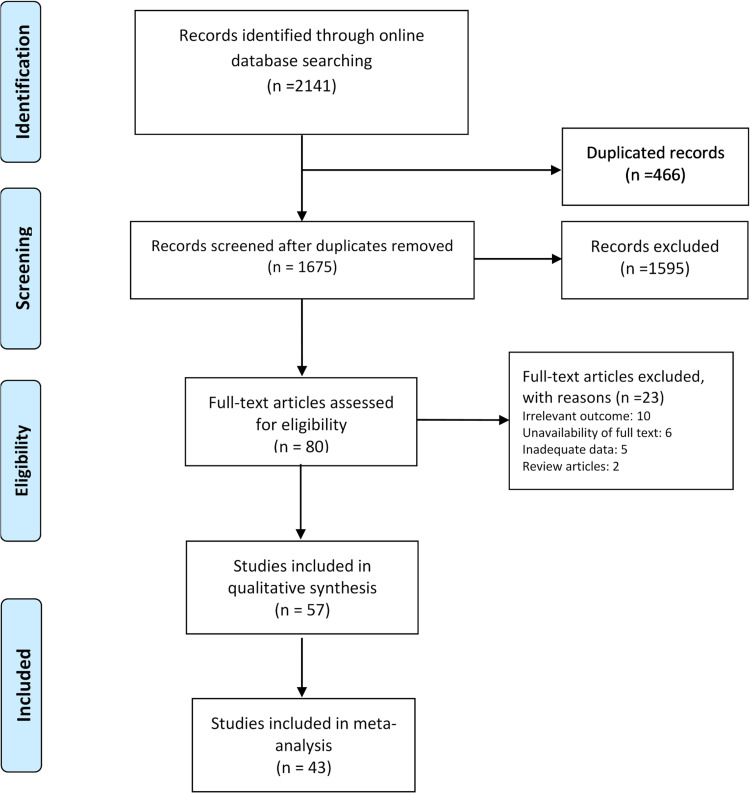
PRISMA flowchart of screened and included studies.

We found a total of 1675 non-duplicate studies, 57 of which were included in the qualitative synthesis and 43 were included in the meta-analysis (Fig. 1). A description of the main characteristics of the included studies is provided in Table 1. The 57 studies included 27 cross-sectional, one case-control, one retrospective cohort and 28 case series/case report studies with sample sizes ranging greatly from one in case-reports to 58 182 for a study in the Hubei province [21]. Inclusion criteria varied greatly across the studies but most study participants were hospitalised patients living or travelling from various provinces in China. Median (range) age of the participants was 46.2 (range: 17 days to 78.5 years) and about 60% were male. Most studies were conducted between January and February 2020. Clinical and epidemiological characteristics of the patients included in the study are presented in Table 2. Among studies that reported exposure history among their participants, most patients were directly or indirectly traced back to the city Wuhan (e.g. lived in Wuhan or had recently travelled to Wuhan) and the Huanan seafood market in Hubei province, China. Several cases of contracting SARS-CoV-2 through close contacts with family members were also reported across the studies. Frequent CT or X-ray findings included thickened texture of the lungs, bilateral focal consolidation, lobar consolidation, ground-glass opacity, patchy consolidation, and unilateral/bilateral pneumonia. Common symptoms reported across the studies included fever, cough, shortness of breath, and fatigue/weakness. Only 15 studies reported some information about the pre-existing conditions of the patients; most of whom had metabolic and cardiovascular underlying conditions.

**Table 1.** Characteristics of the included studies in the systematic review

First author	Publication date (DD-MM-YY)	Study setting/location	Study type	Sample size	Age; mean/range	Male proportion
Chan [22]	24-Jan-20	China, Shenzhen	Case series	6	46.17	0.50
Li [23]	29-Jan-20	China, Wuhan	Cross-sectional	425	55.5	0.56
Chen [1]	30-Jan-20	China, Wuhan	Cross-sectional	99	55.5	0.68
Holshue [24]	31-Jan-20	USA, Washington	Case-report^a^	1	35	1.00
Wu [25]	3-Feb-20	China, Wuhan	Case-report	1	41	1.00
Kim [26]	3-Feb-20	South Korea, Incheon	Case-report	1	35	0.00
Cai [27]	4-Feb-20	China, Shanghai	Case-report^b^	2	7	0.67
Lin [28]	4-Feb-20	China, Jiangxi province	Case-report	2	37	1.00
Backer [29]	6-Feb-20	China, Wuhan	Cross-sectional	88	2 to 72	0.65
Ki [30]	9-Feb-20	South Korea	Cross-sectional	28	42.1	0.54
Jiang [31]	24-Feb-20	China	Cross-sectional	50	NR	NR
Chen [32]	11-Feb-20	China, Wuhan	Case-report	1	1	1.00
Thompson [33]	11-Feb-20	China	Cross-sectional	47	47	0.63
Zhang [34]	11-Feb-20	China, Xiaogan	Case-report	1	0.25 (3 months)	0.00
Duan [35]	12-Feb-20	China, Wuhan	Case-report	1	46	0.00
Stoecklin [36]	13-Feb-20	France, Bordeaux	Case-report	3	36.3	0.67
Huang [37]	15-Feb-20	China, Wuhan	Cross-sectional	41	49.34	0.73
Zhang [14]	15-Feb-20	China, Beijing	Case series	9	35.3	0.56
Lim [38]	17-Feb-20	South Korea, Goyang	Case-report	1	54	1.00
Linton [39]	17-Feb-20	China, multiple cities	Cross-sectional	276	30–59 (>50%)	0.58
COVID-19 Response Team [40]	17-Feb-20	China, multiple cities	Cross-sectional	44 672	30–69 (77.8%)	0.51
Zeng [41]	17-Feb-20	China, Wuhan	Case-report	1	0.046 (17 days)	1.00
Yu [42]	18-Feb-20	China, Shanghai	Case-report	4	74.25	0.50
Xu [43]	18-Feb-20	China, Beijing	Case-report	1	50	1.00
Fang [44]	19-Feb-20	China, Chengdu	Case-report	1	47	1.00
Wang [45]	20-Feb-20	China, Wuhan	Cross-sectional	138	55.3	0.54
Zhu [46]	20-Feb-20	China, Wuhan	Case-report	3	47.33	0.67
Bai [47]	21-Feb-20	China, Anyang	Case series	6	34.75	0.17
Hao [48]	21-Feb-20	China, Shaanxi	Case-report	1	58	1.00
Shi [49]	24-Feb-20	China, Wuhan	Cross-sectional	81	49.5	0.52
Cheng [50]	26-Feb-20	Taiwan, Taoyuan	Case-report	1	55	0.00
Li [51]	26-Feb-20	China, Mainland China	Cross-sectional	44 653	NR	NR
	26-Feb-20	China, Hubei	Cross-sectional	33 366	NR	NR
Yang [52]	26-Feb-20	China, Wenzhou	Cohort	149	45.11	0.54
Shrestha [53]	27-Feb-20	Nepal, Kathmandu	Case-report	1	32	1.00
Tian [54]	27-Feb-20	China, Beijing	Cross-sectional	262	47.5	0.48
Guan [55]	28-Feb-20	China, 30 provinces	Cross-sectional	1099	46.7	0.58
Lillie [56]	28-Feb-20	UK	Case-report	2	36.5	0.50
Wu [57]	29-Feb-20	China, Jiangsu province	Cross-sectional	80	46.1	0.49
Song [58]	1-Mar-20	China	Cross-sectional	11 791	NR	NR
Cheng [59]	2-Mar-20	China, Henan province	Cross-sectional	1079	46.6	0.53
Peng [60]	2-Mar-20	China, Wuhan	Cross-sectional	112	61.3	0.47
Dey [21]	3-Mar-20	China, Hubei province	Cross-sectional	58 182	NR	NR
		China, Other provinces	Cross-sectional	12 264	NR	NR
		Outside of China	Cross-sectional	425	NR	NR
Ruan [61]	3-Mar-20	China, Wuhan	Case control	66	45–75 (>50%)	NR
Young [62]	3-Mar-20	Singapore	Cross-sectional	18	49.5	0.50
Chen [63]	5-Mar-20	China, Guangdong	Case-report	1	46	0.00
Cheng [64]	5-Mar-20	China, Hong Kong	Cross-sectional	42	57.8	0.48
Qiu [65]	5-Mar-20	China, Zhengzhou	Case series	8	25.9	0.50
Rothe [66]	5-Mar-20	Germany, Munich	Case series	5	NR	NR
Spiteri [67]	5-Mar-20	WHO European Region	Cross-sectional	38	41.75	0.66
Wang [68]	5-Mar-20	China, Wuhan	Case-report	2	78.5	0.50
Wang [69]	5-Mar-20	China, Zhengzhou	Cross-sectional	18	41	0.56
Wu [70]	5-Mar-20	China, Tianjin	Cross-sectional	40	44.0	0.33
Yang [71]	5-Mar-20	China	Cross-sectional	325	8 months to 90 years	0.49
Lauer [72]	10-Mar-20	China, outside Hubei province	Cross-sectional	181	44.67	0.60
		Outside mainland China	Cross-sectional	108	NR	NR
Tong [73]	17-May-20^c^	China, Zhoushan	Case-report	3	33	1.00
Arashiro [74]	17-Jun-20^c^	Japan, Cruise ship	Case-report	2	31	0.50
Liu [75]	17-Jun-20^c^	China, Shenzhen	Cross-sectional	365	46.2	0.50

a Studies with a sample size less than or equal to four patients were labelled as case-reports [76].

b A 7-year-old-boy and his parents.

c Studies are in press and will be published in future issues of the respective journals.

**Table 2.** Medical and epidemiological characteristics of the studies included in the systematic review

First author	Exposure history	X-ray/CT findings	Symptoms	Underlying conditions	Incubation period^a^	Time to first clinical visit^b^	Time to ICU admission	Time to recovery	Time to death	Death
Chan [22]	Family cluster; History of travel to Wuhan; No contact with animals; Huanan seafood wholesale market in Wuhan.	14.3% Ground-glass lung opacities; 85.7% Pulmonary infiltrates and multifocal patchy ground-glass opacities, especially around the peripheral parts of the lungs	71.4% Fever; 57.1% Cough; 42.9% Generalised weakness; 14.3% Nasal congestion; 14.3% Sneezing; 14.3% Rhinorrhoea; 14.3% Sore throat; 14.3% Pleuritic chest pain; 28.6% Diarrhoea	28.6% Hypertension; 14.3% Benign intracranial tumour; 14.3% Chronic sinusitis; 14.3% Diabetes	4.5 (0.64)	7.8 (0.74)	NR	NR	NR	NR
Li [23]	11.8% Huanan Seafood Wholesale Market; 4.0% Other wet market but not Huanan Seafood Wholesale Market; 15.3% Contact with another person with respiratory symptoms; 49.9% No exposure to either market or person with respiratory symptoms	NR	NR	NR	5.2 (0.74)	9.7 (0.22)	NR	NR	NR	NR
Chen [1]	49% Huanan seafood market	25% Unilateral pneumonia; 74% Bilateral pneumonia; 14% Multiple mottling and ground-glass opacity	83% Fever; 82% Cough; 31% Shortness of breath; 11% Muscle pain; 9% Confusion; 8% Headache; 5% Sore throat; 4% Rhinorrhoea; 2% Chest pain; 2% Diarrhoea; 1% Nausea and vomiting; 90% More than one sign or symptom; 15% Fever, cough and shortness of breath	51% Chronic medical illness; 40% Cardiovascular and cerebrovascular diseases; 11% Digestive system disease; 13% Endocrine system disease; 1% Malignant tumour; 1% Nervous system disease; 1% Respiratory system disease	NR	NR	NR	NR	NR	11
Holshue [24]	History of travel to Wuhan	Illness day 4: No thoracic abnormalities; Illness day 9: Increasing left basilar opacity	Cough; Fever; Nausea and vomiting	Hypertriglyceridemia	NR	3 (0.0)	NR	15 (0.0)	NR	NR
Wu [25]	Worked at a local indoor seafood market	X-ray: Illness day 6: Abnormal with air-space shadowing such as ground-glass opacities, focal consolidation and patchy consolidation in both lungs; Illness day 11: Bilateral diffuse patchy and fuzzy shadow; CT: Illness day 6: Bilateral focal consolidation; Lobar consolidation and patchy consolidation, especially in the lower lung	Fever; Cough; Sputum production; Dizzy; Weakness; Chest tightness; Dyspnoea	No	NR	6 (0.0)	9 (0.0)	NR	NR	NR
Kim [26]	Living in Wuhan	Initial X-ray: No infiltrations; Illness day 8: Chest infiltrates in the right lower lung field; CT: Illness day 4: Multiple ground-glass opacities in both subpleural spaces	Fever; Chill; Myalgia; Nasal Congestion; Cough; Sputum; Pleuritic chest discomfort; Watery Diarrhoea	Obese (body mass index, 33.4 kg/m^2^)	NR	1 (0.0)	NR	13 (0.0)	NR	NR
Cai [27]	History of travel to Wuhan; Familial contacts	X-ray in child: Thickened texture of both lungs; Blurred right inner lung zone and left posterior region of the heart; Without obvious patch shadows	Fever; Cough; Nasal secretions. Mother of the child: Asymptomatic	NR	NR	2.5 (1.0)	NR	7 (0.0)	NR	NR
Lin [28]	History of travel to Wuhan; Close contact	CT: Patient 1: Multiple regions of patchy consolidation and ground-glass opacities with indistinct border in both lungs; Distributed lesions along the bronchial bundles or within the subpleural lung regions. Patient 2: Focal consolidation along broncho-vascular bundles in right lower lung lobe; Ground-glass opacities in subpleural regions of a left lower lung lobe	Fever; Cough; Throat discomfort	No	NR	2.5 (0.5)	NR	NR	NR	NR
Backer [29]	NR	NR	NR	NR	6.4 (0.54)	NR	NR	NR	NR	NR
Ki [30]	34.6% Arrived from Wuhan; 46.2% Close contact; 3.9% History of travel to Japan; 3.9% History of travel to Thailand; 7.7% Attended a conference in Singapore	NR	Mostly fever; Sore throat; Cough; Chill; Fatigue; Muscle pain	NR	3.9 (0.47) Range: 3–15	NR	NR	13 (0.31)	NR	NR
Jiang [31]	NR	NR	NR	NR	4.9 (0.28)	NR	NR	NR	NR	NR
Chen [32]	Exposed to infection during the consultation	X-ray: Illness day 2: Large blurred images of right upper and lower right lungs; Illness day 7: Partial absorption of right lower lobe pneumonia, right upper lobe atelectasis; CT: Illness day 2: Enhanced texture of lungs; Large consolidating shadows in right lung; Ground glass shadows	Intermittent Diarrhoea; Vomiting; Fever; Shortness of breath; Poor mental response; Lethargy; Poor appetite	NR	NR	1 (0.0)	6 (0.0)	17 (0.0)	NR	NR
Thompson [33]	NR	NR	NR	NR	NR	2.15 (0.43)	NR	NR	NR	NR
Zhang [34]	Unknown	X-ray: Illness day 1: thickened texture of lungs; a small patch-like shadow in the lower right lung field; CT: Illness day 6: Enlarged lung texture	Fever; Cough; Foaming	NR	NR	0 (0.0)	NR	14 (0.0)	NR	NR
Duan [35]	NR	CT: Bilateral and peripheral ground-glass opacities in the superior segments of both lower lobes; Without sparing of subpleural regions.	Fever	NR	NR	7 (0.0)	NR	20 (0.0)	NR	NR
Stoecklin [36]	Arrived from Wuhan	NR	100% Fever; 33.4% Headaches; 100% Cough; 66.7% Fatigue; 33.4% Conjunctivitis; 66.7% Chills	NR	NR	4.34 (1.8)	10 (0.0)	22 (2.0)	NR	NR
Huang [37]	66% Direct exposure to Huanan seafood market	98% Bilateral involvement; CT in ICU patients on admission: Bilateral multiple lobular; Subsegmental areas of consolidation	98% Fever; 76% Cough; 44% Myalgia or Fatigue; 28% Sputum production; 8% Headache; 5% Haemoptysis; 3% Diarrhoea; 55% Dyspnoea	20% Diabetes; 5% Hypertension; 15% Cardiovascular disease; 2% Chronic obstructive pulmonary disease; 2% Malignancy; 2% Chronic liver disease	NR	7 (0.15)	10.5 (0.35)	NR	NR	6
Zhang [14]	66.7% History of travel to Wuhan; 11.1% History of travel to Xiaogan, Hubei; 11.1% Clinician at 3 hospitals in Beijing; 11.1% Familial transmission	X-ray: 22.2% A little exudation of lung; CT: 77.8% Multiple ground glass shadows in the lungs; 33.4% Accompanied by consolidation on the basis of ground glass shadows	88.9% Fever; 55.6% Cough; 44.5% Sore throat; 44.5% Fatigue; 11.1% Nasal congestion; 11.1% Tonsil enlargement; 11.1% Rhinorrhoea	11.12% Diabetes	2.5 (0.65)	4.6 (0.85)	NR	NR	NR	NR
Lim [38]	Living in Wuhan	CT: Small consolidation in the right upper lobe and ground-glass opacities in both lower lobes	Fever; Dry cough; Loose stool; Chilling; Myalgia; Muscle pain	No	NR	3 (0.0)	NR	19 (0.0)	NR	NR
Linton [39]	Direct or indirect exposure to Wuhan and Hubei Province	NR	NR	NR	5.6 (0.33)	6.2 (0.71)	NR	NR	14.5 (1.14)	39
COVID-19 Response Team [40]	68.6% living or going to Wuhan or in close contact with Wuhan patients	NR	NR	NR	NR	NR	NR	NR	NR	1023
Zeng [41]	Family transmission	X-ray: A little right upper lung opacities; CT: No increase in hilar shadows; Enhanced texture of both lungs, and even distribution	Sneezing; Intermittent vomiting; Decreased mental reaction; Milk intake	No	NR	7 (0.0)	NR	13 (0.0)	NR	NR
Yu [42]	Arrived from Wuhan; Family transmission	CT: Patient 1: Interstitial hyperplasia with infection in both of lungs; Chronic bronchitis; Emphysema; Pulmonary bullae of a lingual segment of the left lung; Pulmonary hypertension in both lungs; Increased heart shadow; Calcification of the aorta and aortic wall. Patient 2: 2 Ground-glass opacities on the inferior lobe of the right lung	100% Fever; 25% Poor appetite; 25% Dry cough; 25% Chills	Patient 1: Hypertension, Heart disease; Chronic obstructive pulmonary disease	NR	0.5 (2.9)	1 (0.0)	NR	5 (0.0)	1
Xu [43]	History of travel to Wuhan	X-ray: Illness day 8: Multiple patchy shadows in both lungs; Illness day 12: Progressive infiltrate; Diffuse gridding shadow in both lungs	Fever; Chills; Cough; Fatigue; Shortness of breath	NR	NR	7 (0.0)	NR	NR	14 (0.0)	1
Fang [44]	Family transmission	CT: Ground-glass opacities; Consolidation; or Both in bilateral lungs; ‘Halo sign’ in the basal segment of the lower lobe of the right lung	Cough; Sputum production; Sore throat; Throbbing headache	NR	NR	3 (0.0)	NR	NR	NR	NR
Wang [45]	8.7% were exposed to Huanan Seafood Wholesale Market	CT: 100% Bilateral involvement	98.6% Fever; 69.6% Fatigue; 59.4% Dry cough; 39.9% Anorexia; 34.8% Myalgia; 31.2% Dyspnoea; 26.8% Expectoration; 17.4% Pharyngalgia; 10.1% Diarrhoea; 10.1% Nausea; 9.4% Dizziness; 6.5% Headache; 3.6% Vomiting; 2.2% Abdominal pain	31.2% Hypertension; 14.5% Cardiovascular disease; 10.1% Diabetes; 7.2% Malignancy; 5.1% Cerebrovascular disease; 2.9% COPD; 2.9% Chronic kidney disease; 2.9% Chronic liver disease; 1.4% HIV infection	NR	NR	NR	NR	NR	6
Zhu [46]	Frequently exposed to Huanan Seafood Wholesale Market	CT: Illness day 8: Bilateral fluffy opacities; Illness day 11: Bilateral fluffy opacities in both images, with increased in density, profusion and confluence	Fever; Cough; Chest discomfort	NR	NR	5.5 (1.5)	NR	26.5 (1.5)	14 (0.0)	1
Bai [47]	An asymptomatic carrier arrived from Wuhan; Family transmission	CT: Multifocal ground-glass opacities; Subsegmental areas of consolidation and fibrosis	16% Asymptomatic; Fever; Respiratory symptoms; Sore throat	NR	12.17 (2.06)	NR	NR	NR	NR	NR
Hao [48]	Arrived from Wuhan	CT: At admission: Multiple patchy; Cloud-like high-density shadows in the dorsal segment of the right lower lobe; 4 days after admission: Large ground glass-like high-density shadows on the dorsal segment of the right lung; Patchy cloud-like high-density shadows and consolidation shadows on the left lung	Fever; Sore throat; Fatigue	NR	4 (0.0)	1 (0.0)	NR	NR	NR	NR
Shi [49]	38% Direct exposure to Huanan seafood market; 19% Healthcare workers having close contact with patients; 9% Familial transmission; 35% Without any obvious history of exposure	All patients with abnormal CT imaging features; All lung segments can be involved, and 27% predilection for the right lower lobe; mean number of segments involved: 10.5	73% Fever; 42% Dyspnoea; 22% Chest tightness; 59% Cough; 19% Sputum; 26% Rhinorrhoea; 1% Anorexia; 9% Weakness; 5% Vomiting; 6% Headache; 2% Dizziness; 4% Diarrhoea	11% Chronic pulmonary disease; 12% Diabetes; 15% Hypertension; 4% Chronic renal failure; 10% Cardiovascular disease; 7% Cerebrovascular disease; 5% Malignancy; 9% Hepatitis or Liver cirrhosis	NR	NR	NR	23.2 (0.67)	18.7 (5.7)	3
Cheng [50]	Arrived from Wuhan	X-ray: Progression of prominent bilateral perihilar infiltration; Patchy opacities at bilateral lungs; CT: Persistent multifocal ground-glass opacities with or without superimposed reticulation; Mild fibrotic change at bilateral lungs, including peripheral subpleural regions of both lower lobes; Small irregular opacities	Sore throat; Dry cough; Fatigue; Fever	Hypothyroidism	NR	9 (0.0)	NR	28 (0.0)	NR	NR
Li [51]	NR	NR	NR	NR	NR	NR	NR	NR	NR	1113
	NR	NR	NR	NR	NR	NR	NR	NR	NR	1068
Yang [52]	53.7% Stayed in Wuhan; 3.4% Stay in Hubei province except for Wuhan; 32.9% Contact with people from Hubei province; 10.1% No relation with Hubei province	CT: 3 Involved pulmonary lobes; 6 Involved segments in each patient; Segment 6 and 10 most involved; 2.1% Segments presented ground-glass opacities; 26.8% Segments presented mixed opacity; 7.2% Segments presented consolidation; More localised lesions in the periphery rather than the centre of the lung; More patchy lesions than oval lesions	76.5% Fever; 58.4% Cough; 32.2% Expectoration; 1.3% Dyspnoea; 3.4% Muscle pain; 8.7% Headache; 14.1% Sore throat; 3.4% Snotty; 3.4% Chest pain; 10.7% Chest tightness; 14.1% Chill; 7.4% Diarrhoea; 1.3% Nausea and Vomiting	18.8% Cardio-cerebrovascular disease; 5.4% Digestive system disease; 6.1% Endocrine diseases; 1.3% Malignant tumour; 0.7% Respiratory system diseases; 2.7% Others	NR	6.8 (5.0) Median (IQR)	NR	NR	NR	0
Shrestha [53]	Arrived from Wuhan	NR	Fever	NR	NR	10 (0.0)	NR	14 (0.0)	NR	NR
Tian [54]	40.5% Arrived from; 49.2% Contact with a symptomatic case in the previous 14 days; 67.2% Cluster case	NR	82.1% Fever; 45.8% Cough; 26.3% Fatigue; 6.9% Dyspnoea; 6.5% Headache.	NR	6.7 (5.2) Median (IQR)	4.5 (3.7) Median (IQR)	NR	NR	NR	3
Guan [55]	43.9% Living in Wuhan; 1.9% Contact with wildlife; 31.3% Arrived from Wuhan; 72.3% Contact with Wuhan residents	X-ray: 20.1% Ground-glass opacity; 28.1% Local patchy shadowing; 36.5% Bilateral patchy shadowing; 4.4% Interstitial abnormalities; CT: 56.4% Ground-glass opacity; 41.9% Local patchy shadowing; 51.8% Bilateral patchy shadowing; 14.7% Interstitial abnormalities	88.7% Fever; 0.8% Conjunctival Congestion; 4.8% Nasal congestion; 13.6% Headache; 67.8%% Cough; 13.9% Sore throat; 33.7% Sputum production; 38.1% Fatigue; 0.9% Haemoptysis; 18.7% Shortness of breath; 5.0% Nausea or Vomiting; 3.8% Diarrhoea; 14.9% Myalgia or Arthralgia; 11.5% Chills	1.1% Chronic obstructive pulmonary disease; 7.4% Diabetes; 15.0% Hypertension; 2.5% Coronary heart disease; 1.4% Cerebrovascular disease; 2.1% Hepatitis B infection, 0.9% Cancer, 0.7% Chronic renal disease, 0.2% Immunodeficiency	4 (0.02) Range: 2–7	NR	NR	NR	NR	15
Lillie [56]	Arrived from Wuhan; Close household contact	NR	Fever; Malaise; Dry cough; Sinus congestion; Sore throat; Sinus congestion	NR	NR	3 (1.0)	NR	8.5 (0.5)	NR	NR
Wu [57]	100% Arrived from Wuhan	45.0% Bilateral pneumonia; 23.7% Unilateral pneumonia; 31.2% No abnormal density shadow	78.7% Fever; 63.7% Cough; 37.5% Shortness of breath; 22.5% Muscle pain; 16.2% Headache and mental disorder symptoms; 13.7% Sore throat; 6.1% Rhinorrhoea; 3.7% Chest pain; 1.2% Diarrhoea; 1.2% Nausea and vomiting; 82.5% More than one sign or symptom	31.2% Cardiovascular and cerebrovascular diseases; 6.2% Endocrine system diseases; 3.7% Digestive system disease; 1.2% Respiratory system diseases; 1.2% Malignant tumour; 1.2% Nervous system diseases; 1.25% Chronic kidney disease; 1.2% Chronic liver disease	NR	NR	NR	NR	NR	0
Song [58]	NR	NR	NR	NR	5.01 (0.35)	NR	NR	NR	NR	NR
Cheng [59]	48% Short stay in Wuhan; 35.4% Arrived from Wuhan; 35.4% Close contact; 16.9% No clear case contact history	NR	91.4% Fever; 7.3% Fatigue; 18.2% Cough; 3.1% Sputum; 1.2% Chills; 3.5% Rhinorrhoea; 1.3% Nasal Congestion; 4.0% Dry throat; Sore throat; 3.5% Headache; 1% Chest pain; 3% Shortness of breath; 3.5% Digestive symptoms	NR	NR	NR	NR	NR	NR	19
Peng [60]	NR	NR	90.2 Fever; 67.9% Cough; 63.4% Fatigue or myalgia; 33.9% Chest pain and tightness; 13.4% Diarrhoea; 11.6% Difficulty breathing; 8.9% Stuffy nose; 8.9% Other	20.5% Diabetes; 82.1% Hypertension; 55.4% Coronary heart disease; 35.7% Heart failure	NR	9.84 (0.56)	NR	NR	NR	17
Dey [21]	NR	NR	NR	NR	NR	NR	NR	NR	NR	1696
	NR	NR	NR	NR	NR	NR	NR	NR	NR	69
	NR	NR	NR	NR	NR	NR	NR	NR	NR	5
Ruan [61]	Residents of Wuhan	NR	NR	NR	NR	NR	NR	NR	18.42 (2.27)	
Young [62]	100% History of travel to Wuhan; 5.6% Huanan seafood market; 17% Contact with a healthcare facility in China; 50% Contact with a known case of COVID-19	33% Abnormal chest radiograph finding or lung crepitations; bilateral diffuse airspace opacities; 67% No pulmonary opacities	72% Fever; 83% Cough; 6% Diarrhoea; 12.5% Rhinorrhoea; 61% Sore throat; 11% Shortness of breath	NR	NR	2.39 (0.61)	9 (3)	14.75 (1.22)	NR	0
Chen [63]	History of travel to Wuhan	CT: Multiple patchy ground glass opacities in bilateral subpleural areas	Fever; Sore throat; Cough; Chest distress	NR	NR	7 (0.0)	NR	23 (0.0)	NR	NR
Cheng [64]	33.4% History of travel to mainland China; 4.8% Wet or seafood market; 66.7% Familial transmission	NR	NR	NR	NR	NR	NR	NR	NR	1
Qiu [65]	Arrived from Wuhan; Familial transmission	NR	75% Fever; 37.5% Cough; 12.5% Nasal congestion; 25% Rhinorrhoea; 25% Sneezing; 12.5% Sore throat; 12.5% Tears; 12.5% Diarrhoea; 12.5% Chills; 12.5% Headache; 12.5% Pharyngeal discomfort; 12.5% Rapid heartbeat	NR	9.34 (0.49)	1.75 (0.45)	NR	NR	NR	NR
Rothe [66]	Close contact in workplace	NR	Fever; Sore throat; Myalgia; Chills	NR	4.5 (0.65)	2.75 (0.48)	NR	NR	NR	NR
Spiteri [67]	40% Arrived from China; 60% Infected in Europe	NR	6.4% Asymptomatic; 64.5% Fever; 45.2% Cough; 25.8% Weakness; 16.3% Headaches; 6.4% Sore throat; 6.4% Rhinorrhoea; 6.4% Shortness of breath	NR	NR	3.7 (0.46)	NR	NR	NR	4
Wang [68]	NR	CT: Multiple ground glass Shadow	Intermittent fever; Intermittent cough; Chest tightness; Shortness of breath; Muscle pain	In one patient: coronary heart disease	NR	14.5 (1.5)	NR	NR	NR	NR
Wang [69]	72.2% history of visiting Wuhan; Familial transmission	CT: Ground glass opacities with consolidations	94.4% Fever; 55.6% Cough; 22.2% Shortness of breath; 5.6% Haemoptysis; 11.1% Muscle pain; 5.6% Headache; 5.6% Sore throat; 16.7% Diarrhoea; 5.6% Nausea and vomiting	16.7% Cardiovascular disease; 27.8% Hypertension; 16.7% Diabetes; 11.1% Stroke; 5.6% Malignant tumour	NR	7.75 (0.77)	NR	NR	NR	NR
Wu [70]	Close contact in a department store	NR	95.0% Fever; 35.0% Cough; 27.5% Fatigue; 25.0% Muscle soreness; 15% Diarrhoea; 12.5% Rhinorrhoea; 10% Nasal congestion; 7.5% Headache; Sneezing, Sputum; Nausea; Abdominal pain; 5% Dry mouth; Pharyngeal discomfort; Chest tightness; Asthma; Dizziness; Vomiting	NR	7.25 (0.72)	NR	NR	NR	NR	2
Yang [71]	79% Family transmission; 10% Meals; 6% In a mall or supermarket; 3% Cases of work; 2% Cases of transportation	NR	NR	NR	8.75 (0.26)	NR	NR	NR	NR	NR
Lauer [72]	46.4% Resident of Hubei province; 42.5% History of travel to Wuhan; 11.0% Unknown	NR	NR	NR	5.15 (0.33)	NR	NR	NR	NR	NR
	NR	NR	NR	NR	5.7 (0.66)	NR	NR	NR	NR	NR
Tong [73]	Close contact with a visitor from Wuhan; Familial transmission	NR	Fever; Cough; Skin tingling; Myalgia	NR	NR	4 (2.0)	NR	NR	NR	NR
Arashiro [74]	Close contact	Not clinically significant	Throat dryness and soreness; Throat redness; Slight cough; Fever	NR	NR	4.0 (0.99)	NR	22.5 (0.5)	NR	NR
Liu [75]	43% Close contact; 51% Arrived from Hubei province; 6% Unknown	NR	NR	NR	6.0 (0.70) Range: 1–16	3.89 (0.11)	NR	NR	NR	NR

a Mean (s.e.) day unless specified otherwise.

b Time refers to time from onset of symptoms.

### Mean incubation period

**Fig. 2.**
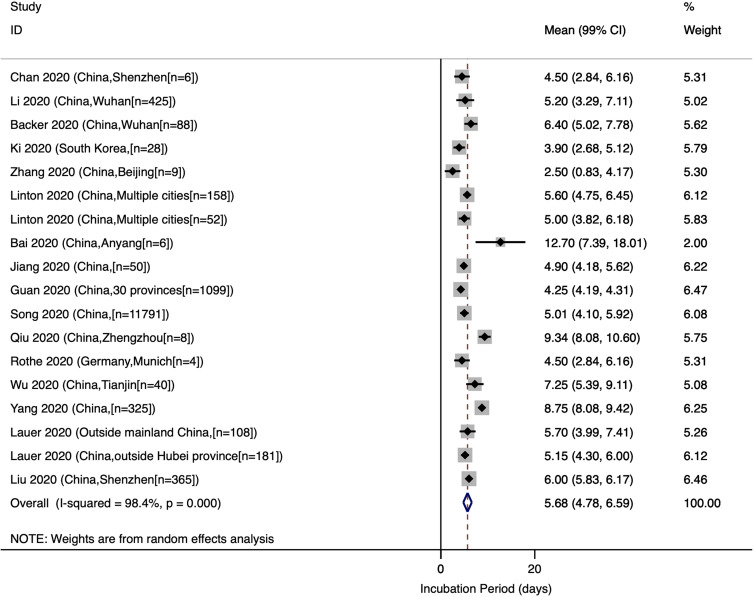
Incubation period of COVID-19.

The estimated mean incubation period obtained from the included studies and the pooled mean are presented in Figure 2. Out of the 18 studies included in the meta-analysis, 15 were conducted in China. The pooled mean incubation period was 5.68 (99% CI: 4.78, 6.59) days. Heterogeneity testing (*I*^2^ = 98.4%) revealed notable differences among the included studies in the meta-analysis. Multivariate meta-regression results showed no significant differences in incubation period time by country (China *vs.* others, Adjusted *β* = 1.76; *P*-value = 0.375), age (Adjusted *β* = −1.16; *P*-value = 0.151) or male percentage of the participants (Adjusted *β* = −12.35; *P*-value = 0.058).

### Mean time from onset of symptoms to first clinical visit

**Fig. 3.**
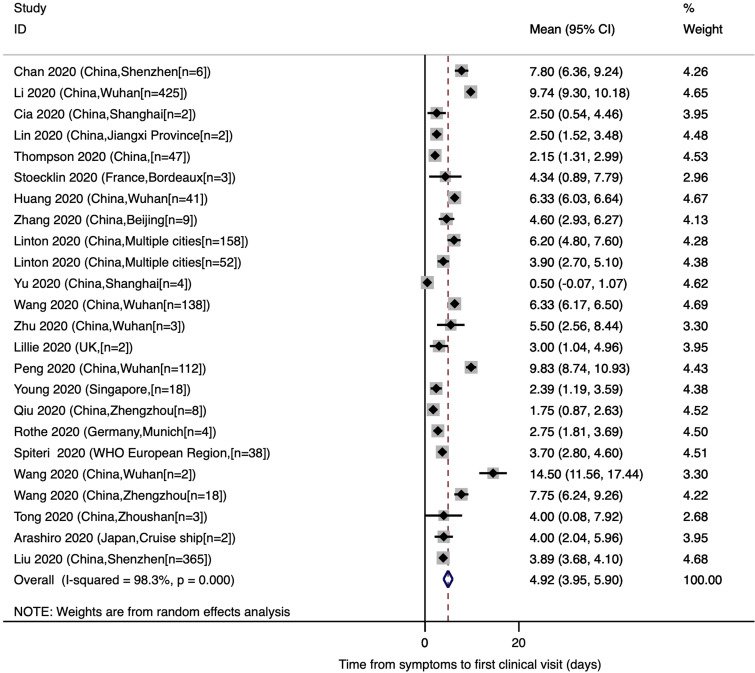
Time from onset of symptoms to first clinical visit for COVID-19 patients.

The estimated mean number of days from the onset of COVID-19 symptoms to first clinical visit was 4.92 (95% CI: 3.95, 5.90). As shown in Figure 3, out of the 24 studies included in the meta-analysis, only six were conducted outside China. Heterogeneity testing (*I*^2^ = 98.3%) revealed notable differences among the included studies in the meta-analysis. Multivariate meta-regression results showed no significant differences in time from onset of symptoms to first clinical visit by country (China *vs.* others, Adjusted *β* = 1.51; *P*-value = 0.411), age (Adjusted *β* = 0.92; *P*-value = 0.153) or male percentage of the participants (Adjusted *β* = −2.60; *P*-value = 0.626).

### Mean time from onset of symptoms to ICU admission

**Fig. 4.**
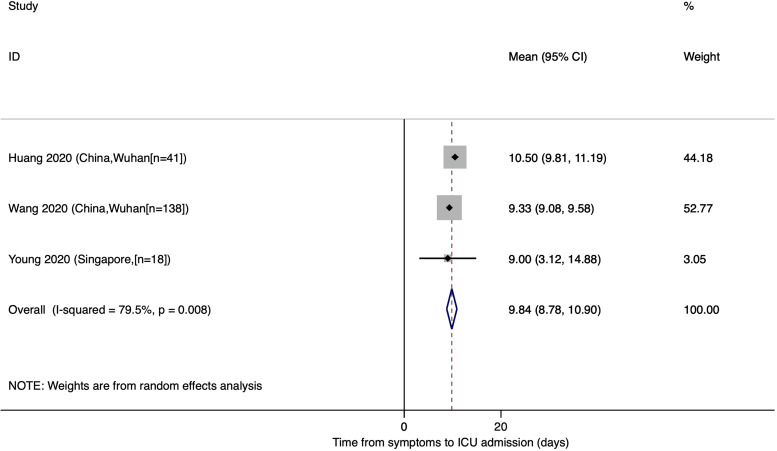
Time from onset of symptoms to ICU admission for COVID-19 patients.

The estimated mean number of days from the onset of COVID-19 symptoms to ICU admission was 9.84 (95% CI: 8.78, 10.90), an estimate that was derived from one study in Singapore and two studies in Wuhan, China (Fig. 4).

### Mean time from onset of symptoms to recovery

**Fig. 5.**
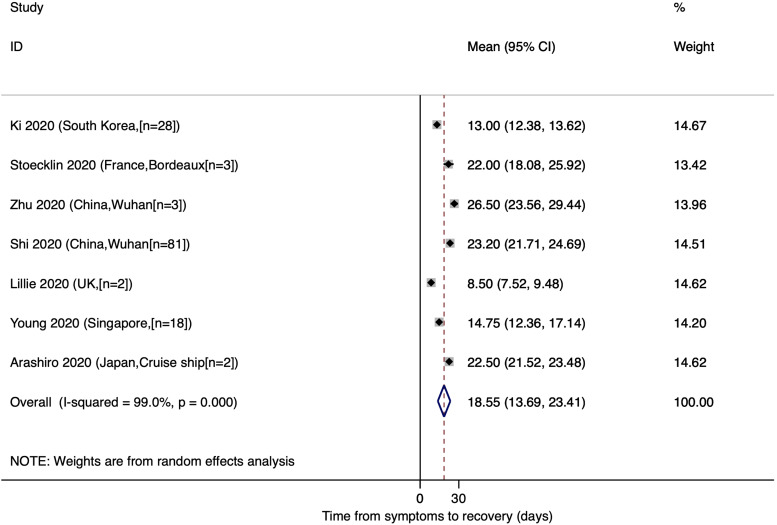
Time from onset of symptoms to recovery for COVID-19 patients.

The estimated mean number of days from the onset of symptoms to recovery was reported in seven studies and the resulting pooled mean was 18.55 (95% CI: 13.69, 23.41). Only two studies were conducted in China and the rest were completed in France, South Korea, the UK, Singapore and Japan (Fig. 5).

### Mean time from onset of symptoms to death

**Fig. 6.**
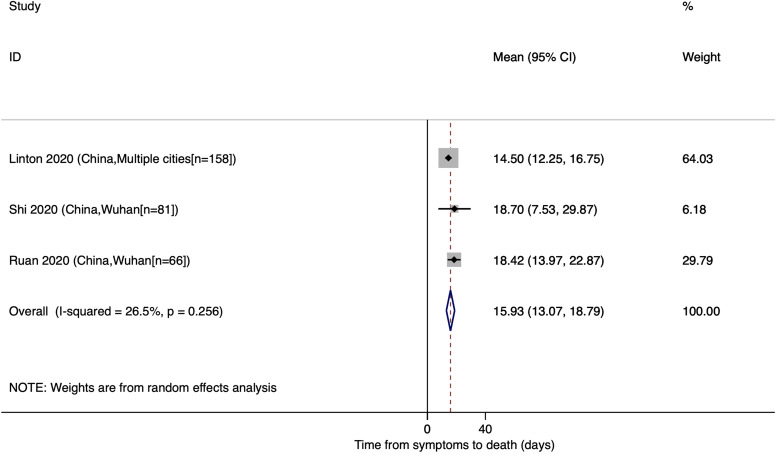
Time from onset of symptoms to death for COVID-19.

The estimated mean number of days from the onset of symptoms to death was reported in three studies with a pooled mean of 15.93 (95% CI: 13.07, 18.79). All of the studies were conducted in China (Fig. 6).

### Case fatality rate

**Fig. 7.**
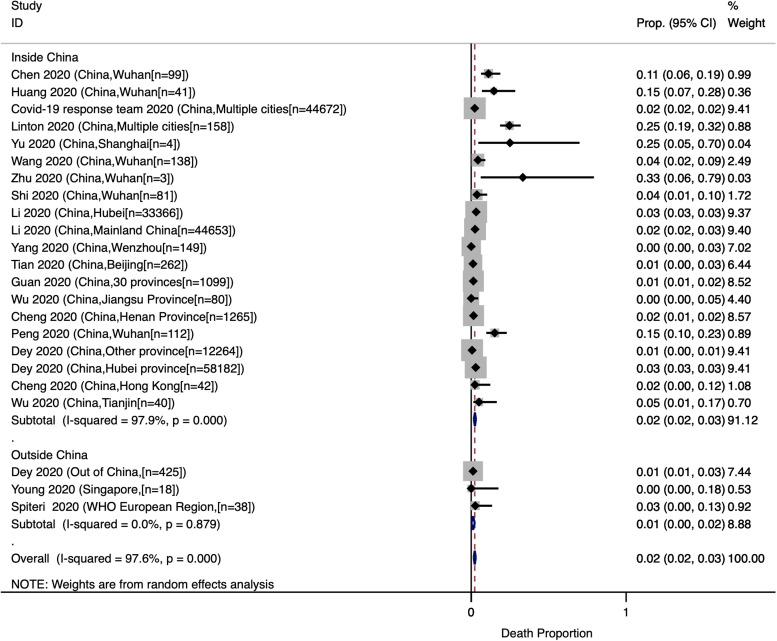
Crude fatality rate among COVID-19 patients.

The estimated CFR among COVID-19 patients was reported in 23 studies; most of which included hospitalised patients, three included ICU patients [37, 45, 60], and none included outpatients. The pooled CFR was estimated as 0.02 (95% CI: 0.02, 0.03) (Fig. 7). Heterogeneity testing (*I*^2^ = 97.6%) revealed notable differences among the included studies in the meta-analysis. Multivariate meta-regression results showed a significant difference in CFR by age (Adjusted *β* = 0.056; *P*-value = 0.003).

## Discussion

We conducted a systematic review and meta-analysis to provide an overview of the epidemiological characteristics of COVID-19 based on the existing evidence as of 11 March 2020. Our findings suggest that COVID-19 has an average incubation period of 5.68 days and there is a lag of 4.92 days from onset of symptoms to the first clinical visit. On average, the symptoms of the patients lasted less than 20 days (18.55 days) before recovery was achieved and the CFR among confirmed COVID-19 patients was 2%, which significantly increased by age. Similar to previous studies [77], fever, dry cough, shortness of breath and fatigue were common symptoms among the patients in the included studies. As expected, history of direct or indirect exposure to Wuhan was frequently reported. The most common radiologic findings were bilateral consolidation and pneumonia [78, 79].

We found the average incubation period of COVID-19 infection to be less than 6 days which is broadly consistent with previously reported estimates [23, 29, 80, 81]. The right tail of the 99% CI of the incubation period for COVID-19 was less than 7 days (6.59). This finding is of particular interest as there are many uncertainties about the incubation period of COVID-19. For example, both the World Health Organization and Centers for Disease Control and Prevention in the USA suggest an incubation period of 2–14 days. However, single outlier cases as long as 19 [47], 24 [15] or 27 days [16] have been reported; estimates that are possibly reflecting a double exposure. Our findings are of particular importance for quarantine-related policies and planning and suggest that the current 14-day quarantine period might be rather conservative. Indeed, we found that except for one small study from China in Anyang city on a cluster of six patients [47], all other studies reported incubation periods less than 10.6 days; therefore, a shorter period of 14 days would suffice and almost all people exposed to SARS-CoV-2 would show symptoms within 11 days of their initial exposure. All in all, decisions to modify or keep the existing policies need to weigh the costs of extending active quarantine against the potential or costs of missing a few patients with delayed-onset symptoms.

COVID-19 seems to have a longer incubation period than that of other acute respiratory viral infections such as human coronavirus (3.2 days), influenza A (1.43–1.64 days), parainfluenza (2.6 days), respiratory syncytial virus (4.4 days) and rhinovirus (1.4 days) [82, 83]. Furthermore, the median incubation period for SARS has been estimated as 4.0 days in 2009 [82], which is considerably lower than what we observed for COVID-19. The longer incubation period of the COVID-19 may be one of the major factors that helps explain its rapid spread in comparison with previous respiratory infection viruses. Other factors contributing to the spread of COVID-19 are the lag between the onset of symptoms and first clinical visit (i.e. 4.92 days) and the high number of asymptomatic COVID-19 patients. These findings suggest that MERS and SARS patients may progress to severe symptoms and respiratory failures [84] much faster than most COVID-19 patients [85].

In comparison to MERS with a fatality rate of 35.67% [86] and SARS with a fatality rate of 11% [87], we found COVID-19 to have a much lower CFR (2%) that significantly increased by age (5.6% increase for every 10-year increase). Although this estimate is comparable with previous studies [40, 88], it is important to recognise the limitations of calculating fatality rates of COVID-19 while the epidemic is still growing. As most COVID-19 patients remain asymptomatic and may recover without seeking medical care, it is likely that the true CFR among people infected with SARS-CoV-2 could be even lower. On the other hand, the estimated fatality rates reported in most studies need to be interpreted with caution as they are often based on the cumulative number of deaths relative to the number of confirmed cases, while patients who die on a given day have been infected at a much earlier date and this would bias the denominator used to calculate the fatality rate [89].

We acknowledge four main limitations of our systematic review. First, our findings are mainly based on studies that recruited patient from clinics and hospitals and therefore, may be biased towards more severe cases. Moreover, our data might be skewed towards early reporting from provinces in China and outcomes might be different in other countries in the Western context. We are, therefore, aiming to update the review as more data become available in the next 12 months to provide more accurate estimates. Second, many studies did not report the study outcomes by subgroups such as age or sex and we could not report group-specific outcomes. Third, we used the mean and the standard error of the incubation period assuming a normal distribution which may have led to the underestimation of the right tail of the distribution. Lastly, given the urgency of the topic and the heterogeneity of the studies included in the review, we did not conduct the risk of bias or quality assessment of the studies. Given the emerging nature of COVID-19 and the observational study design of most of the available evidence, most studies in the review are at a high risk of bias and the quality of existing evidence is relatively low. Nonetheless, our systematic review of literature provides an insightful picture of the epidemiological characteristics of COVID-19 which could inform ongoing public health and public policy decision makings.

## Conclusions

Our findings of the epidemiological characteristics of COVID-19 provide important insight into healthcare systems' prevention and planning efforts. The incubation period (i.e. <11 days in most studies) and the lag between the onset of symptoms and diagnosis (i.e. ~5 days) are longer for COVID-19 compared to other respiratory viral infections including MERS and SARS. Current policies of 14 days of mandatory quarantine for everyone potentially exposed to SARS-CoV-2 might be too conservative and longer quarantine periods might be more justified for extreme cases. As effective vaccination or treatment for COVID-19 are yet to be developed, practising the fundamentals of public health and prevention science such as physical distancing and personal hygiene are critical and need to be emphasised and enforced further to reduce the risk of SARS-CoV-2 transmission.

## Data Availability

All of the data are presented in the paper. The dataset for meta-analysis is available upon reasonable request from the corresponding author (Hamid Sharifi; E-mail: hsharifi@kmu.ac.ir).

## Supplementary material

For supplementary material accompanying this paper visit http://dx.doi.org/10.1017/S0950268820001430. click here to view supplementary material

## References

[1] Chen N, (2020) Epidemiological and clinical characteristics of 99 cases of 2019 novel coronavirus pneumonia in Wuhan, China: a descriptive study. The Lancet 395, 507–513. 10.1016/S0140-6736(20)30211-7 PMC7135076 32007143

[2] World Health Organization (2020) Coronavirus Disease (COVID-19) Pandemic. Geneva: World Health Organization.

[3] Daga MK (2019) From SARS-CoV to coronavirus disease 2019 (COVID-19)-A brief review. Journal of Advanced Research in Medicine 6, 1–9.

[4] Centers for Disease Control and Prevention (2004) SARS Basics Fact Sheet. USA: Centers for Disease Control and Prevention.

[5] World Health Organization. SARS (Severe Acute Respiratory Syndrome). Geneva: World Health Organization.

[6] World Health Organization (2019) Middle East Respiratory Syndrome Coronavirus (MERS-CoV). Geneva: World Health Organization.

[7] World Health Organization (2020) Coronavirus disease 2019 (COVID-19): Situation Report – 51.

[8] Xiao F (2020) Evidence for gastrointestinal infection of SARS-CoV-2. *medRxiv*. 10.1053/j.gastro.2020.02.055 PMC7130181 32142773

[9] Lovelace B, Higgins-Dunn N and Feuer W (2020) WHO considers ‘airborne precautions’ for medical staff after study shows coronavirus can survive in air. USA: CNBC.

[10] CDC COVID-19 Response Team (2020) Severe Outcomes Among Patients with Coronavirus Disease 2019 (COVID-19) — United States, February 12–March 16, 2020. US: Centers for Disease Control and Prevention. 10.15585/mmwr.mm6912e2 PMC7725513 32214079

[11] Picard A (2020) Young people don't get a pass with COVID-19. Canada: The globe and mail.

[12] AlTakarli NS (2020) Emergence of COVID-19 infection: what is known and what is to be expected–narrative review article. Dubai Medical Journal 3, 13–18.

[13] Goh KJ, Kalimuddin S and Chan KS (2020) Rapid progression to acute respiratory distress syndrome: review of current understanding of critical illness from COVID-19 infection. Annals of the Academy of Medicine, Singapore 49, 108–118. 32200400

[14] Zhang MQ (2020) [Clinical features of 2019 novel coronavirus pneumonia in the early stage from a fever clinic in Beijing]. Zhonghua jie he he hu xi za zhi=Zhonghua jiehe he huxi zazhi=Chinese journal of tuberculosis and respiratory diseases 43, E013. 10.3760/cma.j.issn.1001-0939.2020.0013 32061066

[15] Guan W-j (2020) Clinical characteristics of 2019 novel coronavirus infection in China. *MedRxiv*.

[16] Shen S and Woo R (2020) Coronavirus incubation could be as long as 27 days, Chinese provincial government says. UK: Reuters. Feb, 22, 2020.

[17] Moher D (2009) Preferred reporting items for systematic reviews and meta-analyses: the PRISMA statement. Annals of Internal Medicine 151, 264–269. 19622511 10.7326/0003-4819-151-4-200908180-00135

[18] McGowan J (2016) PRESS Peer review of electronic search strategies: 2015 guideline statement. Journal of Clinical Epidemiology 75, 40–46. 27005575 10.1016/j.jclinepi.2016.01.021

[19] Deeks JJ, Higgins JP and Altman DG (2011) Analysing data and undertaking meta-analyses In Higgins JPT and Green S (eds), Cochrane Handbook for Systematic Reviews of Interventions: Cochrane Book Series. UK: The Cochrane Collaboration, pp. 9.1–9.44.

[20] Higgins J and Green S (2011) Cochrane Handbook for Systematic Reviews of Interventions. UK: The Cochrane Collaboration.

[21] Dey SK (2020) Analyzing the epidemiological outbreak of COVID-19: a visual exploratory data analysis (EDA) approach. Journal of Medical Virology 92, 632–638. 32124990 10.1002/jmv.25743 PMC7228278

[22] Chan JF-W (2020) A familial cluster of pneumonia associated with the 2019 novel coronavirus indicating person-to-person transmission: a study of a family cluster. The Lancet 395, 514–523. 10.1016/S0140-6736(20)30154-9 PMC7159286 31986261

[23] Li Q (2020) Early transmission dynamics in Wuhan, China, of novel coronavirus–infected pneumonia. New England Journal of Medicine 382, 1199–1207. 31995857 10.1056/NEJMoa2001316 PMC7121484

[24] Holshue ML (2020) First case of 2019 novel coronavirus in the United States. New England Journal of Medicine 382, 929–936. 32004427 10.1056/NEJMoa2001191 PMC7092802

[25] Wu F (2020) A new coronavirus associated with human respiratory disease in China. Nature 579, 265–269. 32015508 10.1038/s41586-020-2008-3 PMC7094943

[26] Kim JY (2020) The first case of 2019 novel coronavirus pneumonia imported into Korea from Wuhan, China: implication for infection prevention and control measures. Journal of Korean Medical Science 35, e61. 32030925 10.3346/jkms.2020.35.e61 PMC7008073

[27] Cai JH (2020) [First case of 2019 novel coronavirus infection in children in shanghai]. Zhonghua er ke za zhi=Chinese Journal of Pediatrics 58, 86–87. 32102141 10.3760/cma.j.issn.0578-1310.2020.02.002

[28] Lin X (2020) Novel coronavirus pneumonia outbreak in 2019: computed tomographic findings in two cases. Korean Journal of Radiology 21, 365–368. 32056397 10.3348/kjr.2020.0078 PMC7039714

[29] Backer JA, Klinkenberg D and Wallinga J (2020) Incubation period of 2019 novel coronavirus (2019-nCoV) infections among travellers from Wuhan, China, 20–28 January 2020. Eurosurveillance 25, 2000062. 10.2807/1560-7917.ES.2020.25.5.2000062 PMC7014672 32046819

[30] Ki M and Task Force for 2019-nCoV (2020) Epidemiologic characteristics of early cases with 2019 novel coronavirus (2019-nCoV) disease in Republic of Korea. Epidemiology and Health 42: e2020007. 32035431 10.4178/epih.e2020007 PMC7285424

[31] Jiang X, Rayner S and Luo M-H (2020) Does SARS-CoV-2 has a longer incubation period than SARS and MERS? Journal of Medical Virology 95, 476–478. 10.1002/jmv.25708 PMC7166592 32056235

[32] Chen F (2020) [First case of severe childhood novel coronavirus pneumonia in China]. Zhonghua er ke za zhi=Chinese Journal of Pediatrics 58, 179–182. 32135586 10.3760/cma.j.issn.0578-1310.2020.03.003

[33] Thompson RN (2020) Novel coronavirus outbreak in Wuhan, China, 2020: intense surveillance is vital for preventing sustained transmission in new locations. Journal of Clinical Medicine 9, 498. 10.3390/jcm9020498 PMC7073840 32054124

[34] Zhang YH (2020) [2019-novel coronavirus infection in a three-month-old baby]. Zhonghua er ke za zhi=Chinese Journal of Pediatrics 58, E006. 32043842 10.3760/cma.j.issn.0578-1310.2020.0006

[35] Duan YN and Qin J (2020) Pre- and posttreatment chest CT findings: 2019 novel coronavirus (2019-nCoV) pneumonia. Radiology 295, 21. 32049602 10.1148/radiol.2020200323 PMC7233361

[36] Bernard Stoecklin S (2020) First cases of coronavirus disease 2019 (COVID-19) in France: surveillance, investigations and control measures, January 2020. Eurosurveillance 25, 2000094. 10.2807/1560-7917.ES.2020.25.6.2000094 PMC7029452 32070465

[37] Huang C (2020) Clinical features of patients infected with 2019 novel coronavirus in Wuhan, China. The Lancet 395, 497–506. 10.1016/S0140-6736(20)30183-5 PMC7159299 31986264

[38] Lim J (2020) Case of the index patient who caused tertiary transmission of COVID-19 infection in Korea: the application of Lopinavir/Ritonavir for the treatment of COVID-19 infected pneumonia monitored by quantitative RT-PCR. Journal of Korean Medical Science 35, e79–e79. 32056407 10.3346/jkms.2020.35.e79 PMC7025910

[39] Linton NM (2020) Incubation period and other epidemiological characteristics of 2019 novel coronavirus infections with right truncation: a statistical analysis of publicly available case data. Journal of Clinical Medicine 9, 538. 10.3390/jcm9020538 PMC7074197 32079150

[40] Novel Coronavirus Pneumonia Emergency Response Epidemiology Team (2020) [The epidemiological characteristics of an outbreak of 2019 novel coronavirus diseases (COVID-19) in China]. Zhonghua liu xing bing xue za zhi=Zhonghua liuxingbingxue zazhi 41, 145–151.32064853

[41] Zeng LK (2020) [First case of neonate infected with novel coronavirus pneumonia in China]. Zhonghua er ke za zhi=Chinese Journal of Pediatrics 58, E009. 32065520 10.3760/cma.j.issn.0578-1310.2020.0009

[42] Yu P (2020) A familial cluster of infection associated with the 2019 novel coronavirus indicating possible person-to-person transmission during the incubation period. The Journal of Infectious Diseases 22, 1757–1761. 10.1093/infdis/jiaa077 PMC7107453 32067043

[43] Xu Z (2020) Pathological findings of COVID-19 associated with acute respiratory distress syndrome. The Lancet Respiratory Medicine 8, 420–422. 32085846 10.1016/S2213-2600(20)30076-X PMC7164771

[44] Fang X (2020) Changes of CT findings in a 2019 novel coronavirus (2019-nCoV) pneumonia patient. QJM: An International Journal of Medicine 13, 271–272. 10.1093/qjmed/hcaa038 PMC7107315 32073631

[45] Wang D (2020) Clinical characteristics of 138 hospitalized patients with 2019 novel coronavirus–infected pneumonia in Wuhan, China. JAMA 323, 1061–1069. 10.1001/jama.2020.1585 PMC7042881 32031570

[46] Zhu N (2020) A novel coronavirus from patients with pneumonia in China, 2019. New England Journal of Medicine 382, 727–733. 31978945 10.1056/NEJMoa2001017 PMC7092803

[47] Bai Y (2020) Presumed asymptomatic carrier transmission of COVID-19. JAMA 323, 1406–1407. 10.1001/jama.2020.2565 PMC7042844 32083643

[48] Hao W (2020) Clinical features of atypical 2019 novel coronavirus pneumonia with an initially negative RT-PCR assay. Journal of Infection 80, 671–693. 10.1016/j.jinf.2020.02.008 PMC7126654 32092387

[49] Shi H (2020) Radiological findings from 81 patients with COVID-19 pneumonia in Wuhan, China: a descriptive study. The Lancet Infectious Diseases 20, 425–434. 32105637 10.1016/S1473-3099(20)30086-4 PMC7159053

[50] Cheng S-C (2020) First case of coronavirus disease 2019 (COVID-19) pneumonia in Taiwan. Journal of the Formosan Medical Association 119, 747–751. 32113824 10.1016/j.jfma.2020.02.007 PMC7127252

[51] Li Q and Feng W (2020) Trend and forecasting of the COVID-19 outbreak in China. Journal of Infection 80, 469–496. 10.1016/j.jinf.2020.02.014 PMC7154515 32113991

[52] Yang W (2020) Clinical characteristics and imaging manifestations of the 2019 novel coronavirus disease (COVID-19): a multi-center study in Wenzhou city, Zhejiang, China. Journal of Infection 80, 388–393. 32112884 10.1016/j.jinf.2020.02.016 PMC7102539

[53] Shrestha R (2020) Nepal's first case of COVID-19 and public health response. Journal of Travel Medicine 27, taaa024. 32104884 10.1093/jtm/taaa024 PMC7107523

[54] Tian S (2020) Characteristics of COVID-19 infection in Beijing. Journal of Infection 80, 401–406. 32112886 10.1016/j.jinf.2020.02.018 PMC7102527

[55] Guan W-J (2020) Clinical characteristics of coronavirus disease 2019 in China. New England Journal of Medicine 382, 1708–1720. 32109013 10.1056/NEJMoa2002032 PMC7092819

[56] Lillie PJ (2020) Novel coronavirus disease (covid-19): the first two patients in the UK with person to person transmission. Journal of Infection 80, 578–606. 10.1016/j.jinf.2020.02.020 PMC7127394 32119884

[57] Wu J (2020) Clinical characteristics of imported cases of COVID-19 in Jiangsu Province: a multicenter descriptive study. Clinical Infectious Diseases ciaa199. 10.1093/cid/ciaa199 PMC7108195 32109279

[58] Song Q (2020) Study on assessing early epidemiological parameters of coronavirus disease epidemic in China. Zhonghua liu xing bing xue za zhi=Zhonghua liuxingbingxue zazhi 41, 461. 32113196 10.3760/cma.j.cn112338-20200205-00069

[59] Cheng JL (2020) [Epidemiological characteristics of novel coronavirus pneumonia in Henan]. Zhonghua jie he he hu xi za zhi = Zhonghua jiehe he huxi zazhi = Chinese Journal of Tuberculosis and Respiratory Diseases 43, 327–331. 32118390 10.3760/cma.j.cn112147-20200222-00148

[60] Peng YD (2020) [Clinical characteristics and outcomes of 112 cardiovascular disease patients infected by 2019-nCoV]. Zhonghua xin xue guan bing za zhi 48, E004. 32120458 10.3760/cma.j.cn112148-20200220-00105

[61] Ruan Q (2020) Clinical predictors of mortality due to COVID-19 based on an analysis of data of 150 patients from Wuhan, China. Intensive Care Medicine 46, 846–848. 32125452 10.1007/s00134-020-05991-x PMC7080116

[62] Young BE (2020) Epidemiologic features and clinical course of patients infected with SARS-CoV-2 in Singapore. JAMA 323, 1488–1494. 10.1001/jama.2020.3204 PMC7054855 32125362

[63] Chen D (2020) Recurrence of positive SARS-CoV-2 RNA in COVID-19: a case report. International Journal of Infectious Diseases 93, 297–299. 32147538 10.1016/j.ijid.2020.03.003 PMC7129213

[64] Cheng VC (2020) Escalating infection control response to the rapidly evolving epidemiology of the coronavirus disease 2019 (COVID-19) due to SARS-CoV-2 in Hong Kong. Infection Control & Hospital Epidemiology 41, 493–498. 32131908 10.1017/ice.2020.58 PMC7137535

[65] Qiu Y (2020) Epidemiological analysis on a family cluster of COVID-19. Zhonghua liu xing bing xue za zhi=Zhonghua liuxingbingxue zazhi 41, 506. 10.3760/cma.j.cn112338-20200221-00147 32133831

[66] Rothe C (2020) Transmission of 2019-nCoV infection from an asymptomatic contact in Germany. New England Journal of Medicine 382, 970–971. 32003551 10.1056/NEJMc2001468 PMC7120970

[67] Spiteri G (2020) First cases of coronavirus disease 2019 (COVID-19) in the WHO European Region, 24 January to 21 February 2020. Eurosurveillance 25, 2000178. 10.2807/1560-7917.ES.2020.25.9.2000178 PMC7068164 32156327

[68] Wang Y (2020) [COVID-19 complicated with DIC: 2 cases report and literatures review]. Zhonghua xue ye xue za zhi = Zhonghua xueyexue zazhi 41, 245–247. 32133824 10.3760/cma.j.issn.0253-2727.2020.0001 PMC7357925

[69] Wang L (2020) The clinical dynamics of 18 cases of COVID-19 outside of Wuhan, China. European Respiratory Journal 55, 2000398. 32139464 10.1183/13993003.00398-2020 PMC7098482

[70] Wu W (2020) Investigation and analysis on characteristics of a cluster of COVID-19 associated with exposure in a department store in Tianjin. Zhonghua liu xing bing xue za zhi=Zhonghua liuxingbingxue zazhi 41, 489. 32133830 10.3760/cma.j.cn112338-20200221-00139

[71] Yang HY (2020) [The preliminary analysis on the characteristics of the cluster for the Corona virus disease]. Zhonghua liu xing bing xue za zhi=Zhonghua liuxingbingxue zazhi 41, 623–628. 32145716 10.3760/cma.j.cn112338-20200223-00153

[72] Lauer SA (2020) The incubation period of coronavirus disease 2019 (COVID-19) from publicly reported confirmed cases: estimation and application. Annals of Internal Medicine 172, 577–582. 32150748 10.7326/M20-0504 PMC7081172

[73] Tong Z-D (2020) Potential presymptomatic transmission of SARS-CoV-2, Zhejiang Province, China, 2020. Emerging Infectious Diseases 26, 1052–1054. 32091386 10.3201/eid2605.200198 PMC7181913

[74] Arashiro T, Furukawa K and Nakamura A (2020) COVID-19 in 2 persons with mild upper respiratory symptoms on a Cruise Ship, Japan. Emerging Infectious Diseases 26, 1345–1348. 32118533 10.3201/eid2606.200452 PMC7258480

[75] Liu J (2020) Community transmission of severe acute respiratory syndrome coronavirus 2, Shenzhen, China, 2020. Emerging Infectious Diseases 26, 1320–1323. 32125269 10.3201/eid2606.200239 PMC7258448

[76] Murad MH (2018) Methodological quality and synthesis of case series and case reports. BMJ Evidence-Based Medicine 23, 60. 10.1136/bmjebm-2017-110853 PMC6234235 29420178

[77] Ji W (2020) Comparison of severe and non-severe COVID-19 pneumonia: review and meta-analysis. *medRxiv*.

[78] Pormohammad A (2020) Clinical Characteristics, Laboratory Findings, Radiographic Signs and Outcomes of 52,251 Patients with Confirmed COVID-19 Infection: A Systematic Review and Meta-Analysis. *preprintsorg*. 10.1016/j.micpath.2020.104390 PMC7361116 32681968

[79] Lai C-C (2020) Asymptomatic carrier state, acute respiratory disease, and pneumonia due to severe acute respiratory syndrome coronavirus 2 (SARSCoV-2): facts and myths. *Journal of Microbiology*. Immunology and Infection 53, 404–412. 10.1016/j.jmii.2020.02.012 PMC7128959 32173241

[80] Liu T (2020) Transmission dynamics of 2019 novel coronavirus (2019-nCoV). *biorxiv*.

[81] Lauer SA (2020) The incubation period of coronavirus disease 2019 (COVID-19) from publicly reported confirmed cases: estimation and application. Annals of Internal Medicine 172(9), 577–582. 32150748 10.7326/M20-0504 PMC7081172

[82] Lessler J (2009) Incubation periods of acute respiratory viral infections: a systematic review. The Lancet Infectious Diseases 9, 291–300. 19393959 10.1016/S1473-3099(09)70069-6 PMC4327893

[83] Nishiura H and Inaba H (2011) Estimation of the incubation period of influenza A (H1N1-2009) among imported cases: addressing censoring using outbreak data at the origin of importation. Journal of Theoretical Biology 272, 123–130. 21168422 10.1016/j.jtbi.2010.12.017

[84] Hui DS, Memish ZA and Zumla A (2014) Severe acute respiratory syndrome vs. the Middle East respiratory syndrome. Current Opinion in Pulmonary Medicine 20, 233–241. 24626235 10.1097/MCP.0000000000000046

[85] Lai C-C (2020) Severe acute respiratory syndrome coronavirus 2 (SARS-CoV-2) and corona virus disease-2019 (COVID-19): the epidemic and the challenges. International Journal of Antimicrobial Agents 55, 105924. 32081636 10.1016/j.ijantimicag.2020.105924 PMC7127800

[86] Nassar M (2018) Middle East Respiratory syndrome coronavirus (MERS-CoV) infection: epidemiology, pathogenesis and clinical characteristics. European Review for Medical and Pharmacological Sciences 22, 4956–4961. 30070331 10.26355/eurrev_201808_15635

[87] World Health Organization (2003) Consensus Document on the Epidemiology of Severe Acute Respiratory Syndrome (SARS). Geneva, Switzerland: World Health Organization.

[88] World Health Organization (2020) Coronavirus disease 2019 (COVID-19) Situation Report – 30. Geneva, Switzerland.

[89] Baud D, (2020) Real estimates of mortality following COVID-19 infection. The Lancet Infectious Diseases 20(7), 773. 10.1016/S1473-3099(20)30195-X PMC7118515 32171390

