# Supplementary material for: Epidemiological characteristics of COVID-19: a systematic review and meta-analysis
Source: Epidemiol Infect. 2020 Jun 29;148:e130. doi: 10.1017/S0950268820001430 (PMC7343974; doi:10.1017/S0950268820001430)
Supplement: Supplementary file 1 [file S0950268820001430sup001.docx]

*Epidemiology and Infection*

**Epidemiological Characteristics of COVID-19: A Systematic Review and Meta-Analysis [Supplementary Tables]**

Malahat Khalili^†^, Mohammad Karamouzian^†^, Naser Nasiri, Sara Javadi, Ali Mirzazadeh, Hamid Sharifi^*^

† Contributed equally as co-first authors

* Corresponding author: [hsharifi@kmu.ac.ir](mailto:hsharifi@kmu.ac.ir)

| **Supplementary Table S1. PRISMA Checklist** | | | |
| --- | --- | --- | --- |
| **Section/topic** | **#** | **Checklist item** | **Reported on page #** |
| **TITLE** | | |  |
| Title | 1 | Identify the report as a systematic review, meta-analysis, or both. | 1 |
| **ABSTRACT** | | |  |
| Structured summary | 2 | Provide a structured summary including, as applicable: background; objectives; data sources; study eligibility criteria, participants, and interventions; study appraisal and synthesis methods; results; limitations; conclusions and implications of key findings; systematic review registration number. | 2 |
| **INTRODUCTION** | | |  |
| Rationale | 3 | Describe the rationale for the review in the context of what is already known. | 3-4 |
| Objectives | 4 | Provide an explicit statement of questions being addressed with reference to participants, interventions, comparisons, outcomes, and study design (PICOS). | 4 |
| **METHODS** | | |  |
| Protocol and registration | 5 | Indicate if a review protocol exists, if and where it can be accessed (e.g., Web address), and, if available, provide registration information including registration number. | 4 |
| Eligibility criteria | 6 | Specify study characteristics (e.g., PICOS, length of follow-up) and report characteristics (e.g., years considered, language, publication status) used as criteria for eligibility, giving rationale. | 4-5 |
| Information sources | 7 | Describe all information sources (e.g., databases with dates of coverage, contact with study authors to identify additional studies) in the search and date last searched. | 4 |
| Search | 8 | Present full electronic search strategy for at least one database, including any limits used, such that it could be repeated. | Supplement 2 |
| Study selection | 9 | State the process for selecting studies (i.e., screening, eligibility, included in systematic review, and, if applicable, included in the meta-analysis). | 4-5 |
| Data collection process | 10 | Describe method of data extraction from reports (e.g., piloted forms, independently, in duplicate) and any processes for obtaining and confirming data from investigators. | 5 |
| Data items | 11 | List and define all variables for which data were sought (e.g., PICOS, funding sources) and any assumptions and simplifications made. | 5 |
| Risk of bias in individual studies | 12 | Describe methods used for assessing risk of bias of individual studies (including specification of whether this was done at the study or outcome level), and how this information is to be used in any data synthesis. | NR |
| Summary measures | 13 | State the principal summary measures (e.g., risk ratio, difference in means). | 6-7 |
| Synthesis of results | 14 | Describe the methods of handling data and combining results of studies, if done, including measures of consistency (e.g., I^2^) for each meta-analysis. | 6-7 |

| **Supplementary Table S2. Search strategies in databases** |
| --- |
| **Embase** |
| '2019 ncov':ab,ti OR 'sars cov2':ab,ti OR 'covid-19':ab,ti OR 'covid 19':ab,ti OR 'covid 2019':ab,ti OR 'coronavirus disease':ab,ti OR 'novel coronavirus':ab,ti OR 'coronavirus infection':ab,ti OR 'wuhan coronavirus':ab,ti OR '2019 novel coronavirus infection':ab,ti OR '2019-ncov infection':ab,ti OR '2019 novel coronavirus':ab,ti |
| **PubMed** |
| 2019 Novel coronavirus[MeSH] OR 2019-nCoV infection [mesh] OR 2019 novel coronavirus infection [mesh] OR coronavirus disease 2019 virus [mesh] OR wuhan coronavirus [mesh] OR coronavirus [tw] OR Novel coronavirus [tw] OR coronavirus disease [tw] OR covid 2019[tw] OR covid-19[tw] OR sars-cov2 [tw] OR 2019-ncov [tw] |
| **Google Scholar** |
| Coronavirus OR “wuhan coronavirus” OR “coronavirus infection” OR “Novel coronavirus” OR “coronavirus disease” OR “sars-cov” OR covid OR ncov OR *COV* OR COVID-19 |

**Supplementary Table S3. Multivariate meta-regression to assess the sources of heterogeneity on COVID-19 medical and epidemiological characteristics**

|  | Number of studies | Adjusted beta coefficient | P-value |
| --- | --- | --- | --- |
| **Incubation period (in days)** |  |  |  |
| China (vs. other countries) | 11 | 1.76 | 0.375 |
| Age (for every 10 years increase) |  | -1.16 | 0.151 |
| Male (for every 1% increase in male participants) |  | -12.35 | 0.058 |
|  |  |  |  |
| **Time from onset of symptoms to first clinical visit (in days)** |  |  |  |
| China (vs. other countries) | 20 | 1.51 | 0.411 |
| Age (for every 10 years increase) |  | 0.92 | 0.153 |
| Male (for every 1% increase in male participants) |  | -2.60 | 0.626 |
|  |  |  |  |
| **Case fatality rate** |  |  |  |
| China (vs. other countries) | 16 | -0.009 | 0.796 |
| Age (for every 10 years increase) |  | 0.056 | 0.003 |
| Male (for every 1% increase in male participants) |  | 0.095 | 0.341 |
